# Allele specific binding of histone modifications and a transcription factor does not predict allele specific expression in correlated ChIP-seq peak-exon pairs

**DOI:** 10.1038/s41598-023-42637-6

**Published:** 2023-09-20

**Authors:** Claire P. Prowse-Wilkins, Jianghui Wang, Josie B. Garner, Michael E. Goddard, Amanda J. Chamberlain

**Affiliations:** 1 Faculty of Veterinary & Agricultural Science, The University of Melbourne, Parkville, VIC 3010 Australia; 2 Agriculture Victoria, AgriBio, Centre for AgriBiosciences, Bundoora, VIC 3083 Australia; 3 Agriculture Victoria, Ellinbank Dairy Centre, Ellinbank, VIC 3821 Australia

**Keywords:** Epigenomics, Gene regulation

## Abstract

Allele specific expression (ASE) is widespread in many species including cows. Therefore, regulatory regions which control gene expression should show *cis*-regulatory variation which mirrors this differential expression within the animal. ChIP-seq peaks for histone modifications and transcription factors measure activity at functional regions and the height of some peaks have been shown to correlate across tissues with the expression of particular genes, suggesting these peaks are putative regulatory regions. In this study we identified ASE in the bovine genome in multiple tissues and investigated whether ChIP-seq peaks for four histone modifications and the transcription factor CTCF show allele specific binding (ASB) differences in the same tissues. We then investigate whether peak height and gene expression, which correlates across tissues, also correlates within the animal by investigating whether the direction of ASB in putative regulatory regions, mirrors that of the ASE in the genes they are putatively regulating. We found that ASE and ASB were widespread in the bovine genome but vary in extent between tissues. However, even when the height of a peak was positively correlated across tissues with expression of an exon, ASE of the exon and ASB of the peak were in the same direction only half the time. A likely explanation for this finding is that the correlations between peak height and exon expression do not indicate that the height of the peak causes the extent of exon expression, at least in some cases.

## Introduction

Sequence variants that affect complex traits (QTL) are enriched in functional regions of the genome such as promoters, enhancers and transcription factor binding sites^1, 2^ However, the ENCODE project, which has annotated functional elements in multiple human tissues, estimated as much as 80% of the genome is functional so 80% of the genome must still be searched to identify QTL^3^. One mechanism by which a sequence variant might affect a complex trait is by modifying gene expression^4^. These can be identified by associating differences in gene expression with genetic variants (expression QTL, eQTL). *cis* eQTL often have relatively large effects on gene expression but it is still difficult to identify the causal variant due to linkage disequilibrium (LD) between the causal variant and other variants nearby in the genome. *cis* eQTL are also expected to be enriched in functional regions of the genome^5, 6^.

One way to identify functional regions is using a technique called Chromatin Immunoprecipitation followed by sequencing (ChIP-seq) using antibodies for known markers of functional regions such as histone modifications and transcription factor binding^7^. The DNA sequences, when mapped to the genome, define a peak and the height of the peak should indicate the extent of histone modification or binding by a transcription factor. This height has been found to be correlated with the expression of nearby genes^8, 9^. Therefore, *cis* eQTL may affect gene expression by affecting regulatory regions, which would alter peak height so identifying variants that affect the height of ChIP-seq peaks might help to identify eQTL^10^.

It is possible to identify SNPs which are affecting activity at functional regions by detecting a difference in functional markers on homologous chromosomes within the same animal, by counting ChIP-seq reads at heterozygous variants^11^. This is known as allele specific binding (ASB)^12^. Because the reads are in the same animal, differences due to environmental factors and background genotype should be the same at each allele, although there can still be some non-genetic influences such as copy number variants and bias to the reference allele^11, 13^. Similarly, gene expression can show allele specificity (Allele specific expression, ASE)^14^. Although there are few studies for ASB, there have been numerous studies showing widespread ASE in different species, tissues and individuals. For example, the Genotype-Tissue Expression (GTEx) project in humans found that 53% of genes showed ASE in at least one tissue across more than 50 individuals^15^. In these cases, it is assumed that ASE is not driven by the heterozygous SNP but by a variant in a functional region and that the heterozygous SNP in the transcript is just a marker for the difference in allelic expression. The same might be true for allelic differences in ChIP-seq peak height but there is evidence that the causal variant for ASB is likely to occur under the peak it regulates^16^. Therefore, we should be able to find causal variants for eQTL by finding sequence variants that are located under a ChIP-seq peak and which affect the height of the peak. Unfortunately, there are millions of peak-gene pairs and so we experience a multiple testing problem if we test every pair for a possible eQTL. In other words, when we find a sequence variant that affects peak height, we do not know which gene or exon whose expression it might affect.

When comparing tissues, there are many cases where the height of a ChIP-seq peak is correlated with the expression of a gene or exon. That is, tissues with a large ChIP-seq peak have high expression of the gene. If differences in the height of a particular ChIP-seq peak is correlated with the expression of a particular gene, we might expect that a SNP that alters the height of the peak would affect the expression of the linked gene. If this were true, it would reduce the multiple testing problem: that is, for any SNP affecting the height of a particular peak we would know which corresponding gene to examine for an effect of the SNP on gene expression. Then, eQTL for a gene are most likely to be found as SNPs affecting ASB in peaks whose height is correlated with expression of the gene across tissues. Additionally, as most functional regions are thought to act in *cis*, the allele which is higher in the peak should be from the same homologous chromosome as the allele that’s higher in the gene. In this study we describe ASE and ASB in three Holstein dairy cows and their foetal offspring in a total of 22 tissues. Using this data, we test the hypothesis that if the correlation between the height of a ChIP-seq peak and exon expression across tissues is positive, then the allele of a SNP that increases peak height should also increase exon expression.

## Methods

### ChIP-seq and RNA-seq

Tissue sampling and RNA-sequencing for three Holstein dairy cows and two of their foetuses (one male and one female with a shared sire) are described in^17^ and^18^. ChIP-sequencing for all tissues was as described in^16^, with the inclusion of more tissues.

Whole genome sequence for each animal and their sires were available from the 1000 bull genomes project^19^. Average coverage at each base pair of the genome was calculated using the GATK tool DepthOfCoverage^20^. Full sequence genotypes for each of the animals and their sires were phased in taurus Run8 of the 1000 bull genomes project after removing variants with minor allele count < 4, GATK variant recalibration tranche > 99.0 and excessive heterozygous calls (> 0.5) within 500 kb regions showing higher than normal heterozygosity.

To prevent mapping bias a masked genome was created from ARS-UCD1.2^21^ by placing a neutral allele at SNP which were heterozygous in any animal. For example, if a SNP was A/T that SNP was changed to a C in the masked reference genome. ChIP-seq and RNA-seq data was mapped to this masked genome. For the ChIP-seq data, peaks were called for each sample as described in^16^. Consensus peaks were called at each base in the genome which was under a peak in at least two samples.

Alleles at heterozygous genotypes were classified as maternal or paternal based on the genotype of the sire (and the genotype of the dam where available). If the genotype of the sire was homozygous, that allele was classified as paternal in the offspring. In the small number of cases (< 10%) the genotype of the sire was heterozygous, the alleles were classified in the offspring based on the previous SNP genotype, assuming phasing was correct between the two SNPs.

### Allele specific binding and allele specific expression

Maternal and paternal allele counts for each SNP under a consensus ChIP-seq peak or in agene were counted using GATK tools (version 4.1.2^20^). GATK HaplotypeCaller was used to create a gVCF file at base pair resolution. Allele counts were then calculated for each SNP under a peak or in an genewith GenotypeGVCF, using the option “depth per allele by sample”. Allele counts at homozygous SNPs were excluded from analysis, as they were uninformative, as were SNPs with monoallelic read counts as these could be genotyping errors. Only SNP within an exon were used for ASE analysis.

To test whether allele counts from SNPs under the same peak/exon shared paternal or maternal allelic bias, and could be combined, allele counts from SNPs under a peak/exon were compared using a G-test.

For s SNPs under a peak/exon, let:

- n_ij_ = allele count for SNP i where i = 1 to s and j = maternal or paternal.
- n_i._ = total number of counts for SNP i.
- n_.j_ = total number of maternal or paternal alleles over all s SNPs.
- n_.._ = total of all counts.

These make an sX2 contingency table. To test the null hypothesis that the ratio of maternal to paternal alleles is the same for s SNPs.

$$G=2(\sum \left[{n}_{ij}\cdot ln({n}_{ij)}\right]+\sum \left[{n}_{..}\cdot ln({n}_{..})\right]-\sum \left[{n}_{.j}\cdot lnln \left({n}_{.j}\right) \right]-\sum \left[{n}_{i.}\cdot ln({n}_{i.})\right])$$

Following this, maternal allele counts from SNPs under the same peak/exon were summed as were paternal allele counts. Samples with total allele counts less than 10 were excluded from analysis.

Peaks and exons were considered to have ASB or ASE when the allele counts deviated significantly from 1:1 according to a ChI-square value corresponding to p < 0.01 with one degree of freedom.

### Tissue specificity of ASB and ASE

Tissue specificity of ASB and ASE at each feature, was tested statistically with a G-Test.

For s tissues with the feature, let:

- n_ij_ = allele count for SNP i where i = 1 to s and j = maternal or paternal.
- n_i._ = total number of counts for tissue i.
- n_.j_ = total number of maternal or paternal alleles over all s SNPs.
- n_.._ = total of all counts.

These make an sX2 contingency table. To test the null hypothesis that the ratio of maternal to paternal alleles is the same across all tissues

$$G=2(\sum \left[{n}_{ij}\cdot \mathrm{ln}({n}_{ij)}\right]+\sum \left[{n}_{..}\cdot \mathrm{ln}\left({n}_{..}\right)\right]-\sum \left[{n}_{.j}\cdot lnln \left({n}_{.j}\right) \right]-\sum \left[{n}_{i.}\cdot \mathrm{ln}\left({n}_{i.}\right)\right])$$

### Correlation between exon expression and peak height

Read counts for each exon in the GTF file for ARS-UCD1.2.97^21^ were calculated using the featureCounts function of the Subread software package (version 1.5.1^22^) at the exon level, counting read fragments (using the –p option) and allowing for reads to be assigned to more than one exon (with the –O option). Read counts for each consensus peak were also calculated with featureCounts, counting read fragments (using the -p option) with all other settings default. Read counts for both peaks and exons were normalised to counts per million (cpm) in EdgeR (version 3.2.8^23^). The correlation between each exon and each peak within 100 Kb either side of the stranded start site of the exon were calculated across all tissues and animals in R (version 3.6.1). This 100 Kb distance was chosen to identify long-range interactions with a reasonable number of tests.

### Ethics approval and consent to participate

The animal study was reviewed and approved by the Department of Jobs, Precincts, and Regions Ethics Committee (Application No. 2014-23). All methods were carried out in accordance with relevant guidelines and regulations and are reported in accordance with ARRIVE guidelines for the reporting of animal experiments.

## Results

### ChIP-seq and RNA-seq

After filtering for quality (JSD > 0.2) there were 263 ChIP-seq samples across 22 tissues assayed for four histone modifications and one transcription factor (Supplementary Table 1). These were assessed in three lactating Holstein dairy cows and two foetuses. ChIP-seq samples had an average of 108 million filtered mapped reads (Supplementary Table 2).

There were 52 RNA-seq libraries from the same samples (Supplementary Table 1) with an average of 184 million mapped reads (Supplementary Table 2). Further results for the RNA-seq libraries are described in^17, 18^.

### Allele specific binding and allele specific expression

#### SNP calling and filtering

Whole genome sequencing resulted in an average of 14–59 mapped reads per base (Supplementary Table 3) and 35,844,933 SNP called. Only 11–23 million of these SNP were located within a gene or consensus peak (Supplementary Table 4). After allele counting and filtering for homozygous and monoallelic SNPs (and for SNP within exons for ASE) there were an average of 19 thousand SNPs used for ASE analysis and between 400 and 900 thousand SNPs used for ASB analysis (Supplementary Table 4).

#### Combining SNPs in the same feature

To test whether SNPs under the same peak or exon could be combined, the direction of bias (either bias towards the maternal or paternal allele) of allele counts at heterozygous SNPs within the same feature (exon or peak) was tested using a G test. The number of features that had significant (p < 0.05) differences in allelic bias between SNPs in the same feature was only slightly more than expected at this p-value (Supplementary Table 5). Therefore, there is only a difference in the direction of allelic bias between SNPs from the same feature in a small number of features, so combining allele counts from SNPs in the same feature should accurately reflect the direction of bias in that feature in most cases.

#### ASB and ASE per peak/exon

ASB and ASE were tested at each feature individually within each sample. In total ~ 40 thousand exons were tested and ~ 400 thousand peaks (Table 1). Between 41 and 55% of peaks (depending on the functional mark tested) had significant ASB in at least one sample and 69% of exons had significant ASE in at least one sample. Not all peaks/exons were able to be tested for ASB/ASE (Table 1).

**Table 1:** The number of consensus peaks and exons as well as the number of these features (peaks or exons) tested and the number of features with significant allelic bias in at least one sample.

Feature	Number of consensus peaks or exons	Number of features tested	Number of features significant in at least one sample (p < 0.001)
Exons	239,017	40,089	21,834 (54%)
H3K4Me3	1,325,868	429,650	87,293 (20%)
H3K4Me1	772,438	407,294	128,466 (32%)
H3K27Me3	799,109	388,044	93,560 (24%)
H3K27ac	1,230,402	433,175	119,677 (28%)
CTCF	1,385,329	460,520	101,848 (22%)

#### Tissue specificity of ASB and ASE

**Figure 1:**
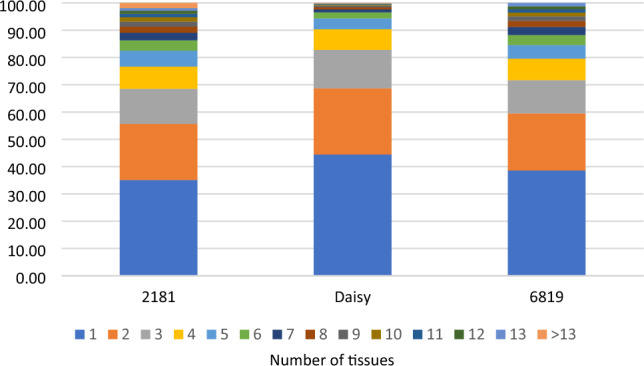
The percentage of exons with significant ASE which were significant in one or multiple tissues, where greater than one tissue tested.

For the three adult cows tested, exons with significant ASE in each cow were tested to see how often the same exon was significant in multiple tissues within the same cow. Although the majority of significant exons were tested in multiple tissues, a large proportion of these were only significant in one or few tissues (Fig. 1).

**Figure 2:**
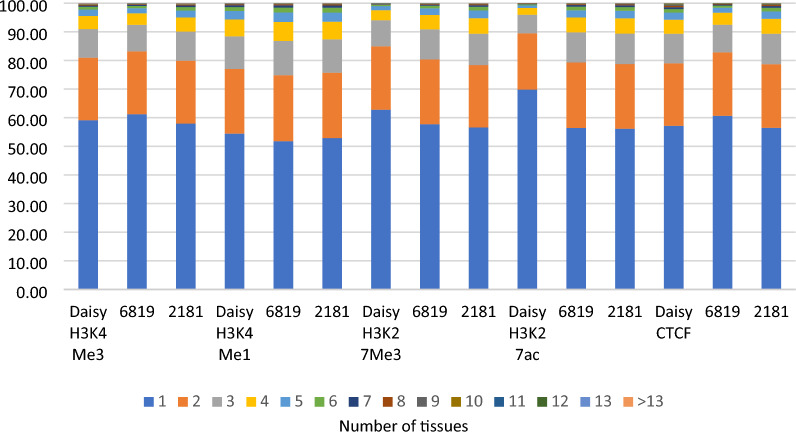
The percentage of peaks with significant ASB which were significant in one or multiple tissues, where greater than one tissue tested.

For the three adult cows tested, peaks with significant ASB in each cow were tested to see how often the same peak was significant in multiple tissues within the same cow. Although the majority of significant peaks were tested in multiple tissues, a large proportion of these were only significant in one or two tissues (Fig. 2).

The significance of the differences in ASB or ASE between tissues was tested using a G Test to compare allele counts per feature across the tissues. In more than 70% of significant features which were tested in multiple tissues, the allele ratio varied significantly (p < 0.05), suggesting widespread differences between tissues in ASE or ASB (Table 2).

**Table 2:** Percentage of features (peaks or exons), which were significant in one tissue, with significantly different allele counts across the tissues it was tested in.

	Cow	Number of significant features tested in > 1 tissue	Number of significant features where bias was different between tissues p < 0.05 (% of total significant SNP)
ASE	2181	12,209	9,386 (77%)
	6819	11,389	8,391 (74%)
	Daisy	9,526	8,143 (85%)
ASB-H3K27ac	2181	86,939	67,295 (77%)
	6819	101,520	81,341 (80%)
	Daisy	52,259	36,671 (70%)
ASB-H3K27Me3	2181	77,469	56,510 (73%)
	6819	71,576	53,160 (74%)
	Daisy	61,918	43,870 (71%)
ASB-H3K4Me3	2181	76,148	57,526 (76%)
	6819	63,814	47,967 (75%)
	Daisy	73,078	54,505 (75%)
ASB-H3K4Me1	2181	83,432	64,003 (77%)
	6819	104,166	81,878 (79%)
	Daisy	81,769	62,708 (77%)
ASB-CTCF	2181	82,097	64,923 (79%)
	6819	79,220	61,915 (78%)
	Daisy	79,108	61,926 (78%)

Although these peaks and exons were significant in only one tissue, it is possible that the direction of bias is the same in the other tissues but there are not enough allele counts to reach significance. We tested whether the direction of bias in features significant in one tissue, was the same in the other tissues the feature was tested in. The majority of exons significant for ASE in one tissue showed the same direction of bias across 80–100% (33%) or 60–80% (31%) of tissues tested (Table 3). For the peaks significant for ASB ~ 40% displayed the same bias in 60% or more of the tissues the peaks were tested in, but ~ 40% showed roughly 50–50 bias in either direction.

**Table 3:**
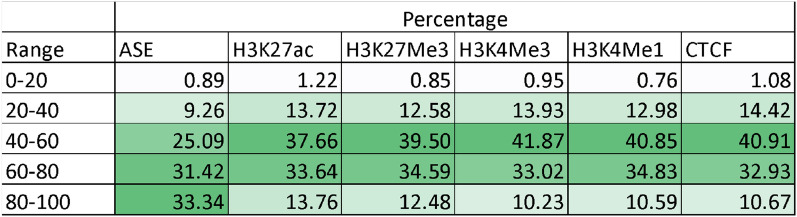
For features significant in one tissue, the percentage of times the direction of effect was the same in 0–20,20–40,40–60,60–80 and 80–100% of the other tissues, averaged across three adult cows.

#### Correlation between exon expression and peak height

Expression for each exon was tested for correlation with peak height of each peak within 100 Kb either side of the exon start position. There were substantially more significant correlations than expected by chance (> 14% of peak-exon pairs tested were correlated, Table 4). Most correlations were positive, that is high peak height was associating with high gene expression, except for H3K27Me3 where 53% of correlations were negative.

**Table 4:** The number of peak-exon pairs tested for correlation for each mark as well as the number of significant correlations and the percentage of significant correlations which were positive or negative.

	Number of peak-exon pairs tested	Number significant p < 0.05 (% of total)	Positive correlations (%)	Negative correlations (%)
H3K4Me3	23,660,487	3,318,175 (14%)	64	36
H3K4Me1	9,637,795	1,596,160 (17%)	64	36
H3K27Me3	10,649,744	1,537,563 (14%)	47	53
H3K27ac	20,614,498	3,341,009 (16%)	76	24
CTCF	23,413,678	3,643,158 (16%)	58	42

#### Direction of ASB and ASE in correlated peak-exon pairs

**Figure 3:**
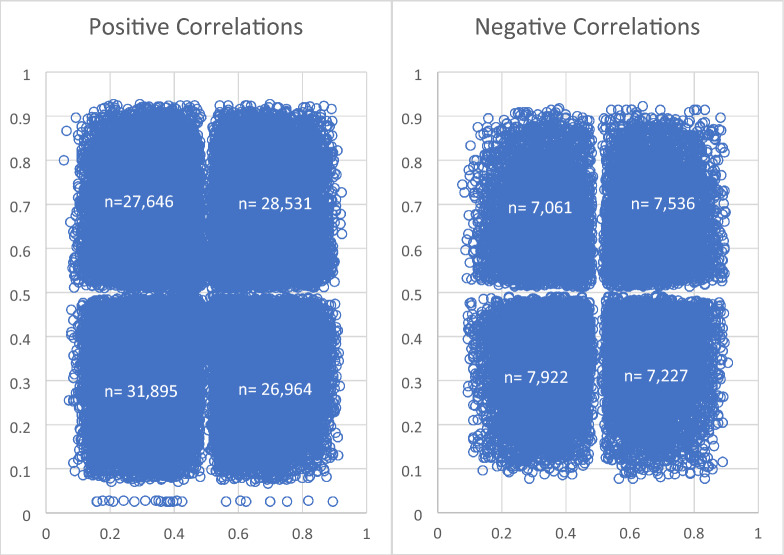
Scatter plot of the proportion of the total allele counts which were maternal in the H3K27ac peak (x-axis) and the exon it was correlated with for all positively correlated peak-exon pairs (left) and negatively correlated peak-exon pairs (right). Each dot represents one sample.

For each significantly correlated exon-peak pair, the ASE and ASB ratio was compared across all samples where ASE and ASB were both significant at p < 0.05. We expected that for positive correlations the direction of ASE and ASB in the exon and peak would be the same in the majority of cases (i.e. when the count from the maternal allele was higher than the paternal allele in the peak, it would also be higher in the exon) and for the negative correlations the direction would be the reverse. However, we found that there were roughly equal cases of ASE and ASB having the same or different directions across all tissues and correlated peak-exon pairs, regardless of direction of correlation (Fig. 3 and Table 5). We also tested the proportion of samples with the same direction of ASB and ASE in peak-gene pairs correlated across smaller distances in H3K27ac with similar results (Supplementary Table 6).

**Table 5:** The total number of samples which had ASE and ASB compared for each mark and the total number of samples where the ASE and ASB had the same direction.

	Correlation	Total samples compared	Total samples with same direction in peak and exon
H3K27ac	Positive	115,036	60,426 (52.5%)
	Negative	29,746	15,458 (51.9%)
H3K27Me3	Positive	20,145	10,023 (49.8%)
	Negative	35,095	17,305 (49.3%)
H3K4Me3	Positive	70,543	38,088 (54%)
	Negative	37,436	19,723 (52.7%)
H3K4Me1	Positive	63,591	32,684 (51.4%)
	Negative	37,949	19,454 (51.3%)
CTCF	Positive	82,194	41,525 (50.5%)
	Negative	54,762	27,725 (50.6%)

To be compared the peak-exon pair had to be significantly positively or negatively correlated at p < 0.05 and the ASE and ASB in each sample had to be significant at p < 0.05.

## Discussion

The reported extent of ASE and its tissue specificity largely depends on the power of the experiment. Chamberlain et al.^17^ previously reported ASE in a single dairy cow (also tested here) across multiple tissues using different methods to those we used. They reported a slightly higher number of genes were significant for ASE (74–89%) than reported here (54% of exons, Table 1). Also in cattle^24^, found that 13% of genes displayed ASE, however this was in a single tissue^17^. reported that 94% of genes with ASE only displayed ASE in a subset of tissues and^25^ (in mice) found that 82% of ASE genes were specific to one of two tissues. We found that 77% of exons with significant ASE showed statistically significant heterogeneity in ASE between tissues (Table 2). In half the exons with significant ASE, only 2 tissues were significant for ASE in that exon (Fig. 1). However, for 64% of exons with significant ASE, > 60% of tissues showed the same direction of ASE (Table 3), even though they were not all significant. Therefore, we confirmed that ASE is widespread and although the extent of ASE varies between tissues, the direction of ASE is the same in many tissues.

There are few studies for ASB in histone modifications, however^26^, found that up to 30% of heterozygous sites tested in human lymphoblastoid cell lines showed ASB in the same marks we have described here. This is similar to our study which found that 22–32% of peaks showed ASB in a larger number of tissues. Studies for ASB in CTCF^27^ report lower proportions of ASB in heterozygous sites in CTCF peaks (11%) than reported here (22% of peaks Table 1) again potentially due to the larger numbers of tissues investigated in our study. Like ASE, we found statistically significant heterogeneity between tissues in ASB (Table 2) and even higher rates of tissue specific ASB with between 50 and 70% of peaks significantly ASB in only one tissue (Fig. 2). However, for ~ 45% of peaks with significant ASB, the direction of ASB in other tissues was the same in > 60% of the tissues where the peak was tested (Table 3). Therefore, like ASE, ASB is widespread in the genome but appears to be slightly more tissue specific than ASE.

A disadvantage of using SNPs to identify difference in binding and expression from homologous chromosomes is that ASE and ASB are only able to be detected when there are SNPs within the feature of interest and when these SNP are heterozygous. In our study, peaks or exons which do not have SNPs, or which only have SNP which are homozygous in all 5 samples were unable to be analysed (Table 1). Therefore, the results presented here are a lower bound of differential expression and binding in the bovine genome. Future work can address this limitation by increasing the sample size of the study.

Previous work^16^ found 6–11% of peak-gene pairs (all peaks within 100 Kb of the TSS of a gene) were correlated across tissues, identifying thousands of peaks potentially linked to differences in gene expression. In this study, we increased the number of tissues and individuals tested, and tested exon counts rather than gene counts. This resulted in up to 17% of peak-exon pairs with a significant correlation (Table 4). As expected, the vast majority (74%) of the correlations with the activating mark H3K27ac were positive, indicating high H3K27ac peak height was correlated with high exon expression as seen in other studies^28^. This was also observed in H3K4Me1 and H3K4Me3. Interestingly H3K27Me3, a repressor mark, only had slightly more negative correlations than positive. Similarly, CTCF had almost equal numbers of positive and negative correlations which is consistent with other studies^27^.

If functional marks are truly regulating gene expression and this regulation is in *cis*, then the allele specific bias observed in a functional mark should be mirrored in the exon expression bias from the same chromosome. That is, if there is a higher H3K27ac peak on the maternal chromosome than on the paternal chromosome, we should observe higher exon expression from the maternal chromosome than from the paternal chromosome. To detect this, we selected peak-exon pairs with positive correlations and compared the direction of significant ASE and ASB in these peaks and exons across all tissues and animals. Just over 50% of the time the direction of ASB and ASE was the same, indicating there was no or little correlation between ASB and ASE other than that expected by chance (Fig. 3 and Table 5). A possible explanation for this is that the correlation between peak height and exon expression seen across tissues is not causal. For instance, both might be affected by the concentration of a transcription factor which affects the height of many peaks, although most of these peaks have no effect on expression of the exon. Other potential explanations could be that our classification of alleles as maternal and paternal may be incorrect, or the regulation of genes (from functional regions) may be in *trans* rather than *cis* and there is a post-transcriptional mechanism to explain ASE in the transcripts from the exon in the cytoplasm.

There have been multiple studies showing differential expression of genes from homologous chromosomes is widespread^14, 15^. Additionally, non-coding functional regions are thought to regulate gene expression in *cis*. Therefore, it stands to reason that non-coding functional regions should also display allele specificity and that the direction of allele specificity in the non-coding functional region and the gene it is regulating should be the same. This paper confirmed that ASE and ASB are widespread in the genome but did not find that the direction of ASE and ASB could be predicted from the correlation of peak height and exon expression across tissues. This work reinforces that correlations between peak height and gene expression may not always imply a causal relationship.

### Supplementary Information

Supplementary Table 1.

Supplementary Table 2.

Supplementary Tables.

## Data Availability

The datasets analysed for this study are publicly available. RNA-seq data is available here: https://www.ebi.ac.uk/ena/browser/view/PRJEB35127 and here: https://www.ebi.ac.uk/ena/browser/view/PRJEB25677. ChIP-seq data is available here: https://www.ebi.ac.uk/ena/browser/view/PRJEB53044 and here: https://www.ebi.ac.uk/ena/browser/view/PRJEB41939. Detailed accession data is available in Supplementary Table 2.
