# Supplementary material for: Allele specific binding of histone modifications and a transcription factor does not predict allele specific expression in correlated ChIP-seq peak-exon pairs
Source: Sci Rep. 2023 Sep 20;13:15596. doi: 10.1038/s41598-023-42637-6 (PMC10511416; doi:10.1038/s41598-023-42637-6)
Supplement: Supplementary file 1 — Supplementary Table 1. [file 41598_2023_42637_MOESM1_ESM.docx]

***Supplementary Table 1. The number of samples assayed for each histone mark, transcription factor or gene expression (RNA).***

| **Tissue** | **CTCF** | **H3K27ac** | **H3K27Me3** | **H3K4Me3** | **H3K4Me1** | **RNA** |
| --- | --- | --- | --- | --- | --- | --- |
| **Adrenal Cortex** | 2 | 2 | 2 | 2 | 2 | 2 |
| **Adrenal Gland** | 1 | 1 | 1 | 1 | 1 | 1 |
| **Adrenal Medulla** | 2 | 2 | 2 | 2 | 2 | 2 |
| **Foetal Heart** | 2 | 2 | 0 | 2 | 2 | 2 |
| **Foetal Kidney** | 2 | 2 | 2 | 2 | 2 | 2 |
| **Foetal Liver** | 2 | 2 | 2 | 2 | 2 | 2 |
| **Foetal Lung** | 2 | 2 | 2 | 2 | 2 | 2 |
| **Heart** | 3 | 3 | 3 | 3 | 3 | 3 |
| **Intestinal Lymph Node** | 3 | 3 | 3 | 3 | 3 | 3 |
| **Kidney** | 3 | 2 | 2 | 3 | 3 | 3 |
| **Liver** | 3 | 3 | 3 | 3 | 3 | 3 |
| **Lung** | 3 | 2 | 2 | 3 | 3 | 3 |
| **Mammary Gland** | 3 | 3 | 3 | 3 | 3 | 3 |
| **Ovary** | 2 | 3 | 3 | 3 | 3 | 3 |
| **Pancreas** | 2 | 2 | 3 | 3 | 3 | 0 |
| **Placenta** | 2 | 2 | 2 | 2 | 2 | 2 |
| **Skin Black** | 2 | 2 | 2 | 2 | 2 | 2 |
| **Skin White** | 2 | 1 | 2 | 2 | 2 | 2 |
| **Spleen** | 3 | 3 | 3 | 3 | 3 | 3 |
| **Thymus** | 3 | 3 | 3 | 3 | 3 | 3 |
| **Thyroid** | 3 | 3 | 3 | 3 | 3 | 3 |
| **Tongue** | 1 | 3 | 3 | 3 | 3 | 3 |
