# Supplementary material for: Allele specific binding of histone modifications and a transcription factor does not predict allele specific expression in correlated ChIP-seq peak-exon pairs
Source: Sci Rep. 2023 Sep 20;13:15596. doi: 10.1038/s41598-023-42637-6 (PMC10511416; doi:10.1038/s41598-023-42637-6)
Supplement: Supplementary file 3 — Supplementary Tables. [file 41598_2023_42637_MOESM3_ESM.docx]

***Supplementary Table 3. Details of whole genome sequence data used for SNP calling.***

| **Animal** | **Mapped reads** | **% properly paired** | **% duplicates** | **Coverage** |
| --- | --- | --- | --- | --- |
| 6819-Fetus | 396,177,062 | 89.10% | 3.50% | 16.04 |
| 6819 | 375,213,487 | 87.60% | 3.20% | 15.15 |
| Daisy | 2,305,337,589 | 87.50% | 11.80% | 59.09 |
| 2181 | 462,431,673 | 87.70% | 3.70% | 18.3 |
| 2181-Fetus | 366,631,205 | 88.30% | 3.50% | 14.71 |

***Supplementary Table 4. The number of SNP found in each feature and the number of SNP used for allele specific analysis after filtering for homozygous and monoallelic SNP and, for ASE, for SNP only in exons.***

| **Feature** | **Number of SNP in feature** | **Average number of SNP after filtering** |
| --- | --- | --- |
| Genes | 12,815,284 | 19,566 (exons only) |
| H3K4Me3 | 11,380,825 | 476,686 |
| H3K4Me1 | 23,732,706 | 978,450 |
| H3K27Me3 | 23,402,907 | 811,525 |
| H3K27ac | 13,662,602 | 543,172 |
| CTCF | 13,042,640 | 556,408 |

***Supplementary Table 5. The number of features (peaks or exons) which had multiple heterozygous SNPs within them and the number of times these SNPs displayed different (P < 0.05) bias.***

| Feature | Number of features | Number of features with multiple SNPs in at least one sample | Number of features with significantly different allelic bias at SNPs in the feature  (P < 0.05) | Percentage different (of total features) |
| --- | --- | --- | --- | --- |
| Exons | 42,306 | 13,514 | 5,889 | 14% |
| H3K4Me3 | 488,435 | 256,170 | 19,232 | 4% |
| H3K4Me1 | 448,291 | 306,065 | 57,371 | 12% |
| H3K27Me3 | 441,584 | 293,397 | 33,693 | 7% |
| H3K27ac | 489,151 | 275,554 | 41,739 | 8% |
| CTCF | 525,208 | 289,892 | 32,698 | 6% |

***Supplementary Table 6. The percentage of correlated H3K27ac peak-gene pairs which had the same direction of ASB and ASE across differing distances. To be compared the peak-exon pair had to be within the distance specified from each other, be significantly positively or negatively correlated at p<0.05 and the ASE and ASB in each sample had to be significant at P < 0.05.***

| **Positive Correlations** | | **Negative Correlations** | |
| --- | --- | --- | --- |
| **Distance (Kb)** | **% Same direction** | **Distance (Kb)** | **% Same direction** |
| 100 | 52.5 | 100 | 51.9 |
| 50 | 53.25 | 50 | 52.77 |
| 30 | 53.39 | 30 | 53.59 |
| 10 | 54.78 | 10 | 54.39 |
| 5 | 56.16 | 5 | 54.17 |
| 1 | 55.17 | 1 | 55.17 |
